# Effect of infrared radiation on the lens

**DOI:** 10.4103/0301-4738.77010

**Published:** 2011

**Authors:** Eman Mohamed Aly, Eman Saad Mohamed

**Affiliations:** Department of Basic Science, Biophysics and Laser Science Unit, Research Institute of Ophthalmology, Giza, Egypt

**Keywords:** Electrophoresis, Fourier transform infrared spectroscopy, infrared, lens, protein, rabbit

## Abstract

**Background:**

Infrared (IR) radiation is becoming more popular in industrial manufacturing processes and in many instruments used for diagnostic and therapeutic application to the human eye.

**Aim:**

The present study was designed to investigate the effect of IR radiation on rabbit’s crystalline lens and lens membrane.

**Materials and Methods:**

Fifteen New Zealand rabbits were used in the present work. The rabbits were classified into three groups; one of them served as control. The other two groups were exposed to IR radiation for 5 or 10 minutes. Animals from these two irradiated groups were subdivided into two subgroups; one of them was decapitated directly after IR exposure, while the other subgroup was decapitated 1 hour post exposure. IR was delivered from a General Electric Lamp model 250R 50/10, placed 20 cm from the rabbit and aimed at each eye. The activity of Na^+^-K^+^ ATPase was measured in the lens membrane. Soluble lens proteins were extracted and the following measurements were carried out: estimation of total soluble protein, sodium dodecyl sulfate-polyacrylamide gel electrophoresis (SDS-PAGE) and Fourier transform infrared (FTIR) spectroscopy. For comparison between multiple groups, analysis of variance was used with significance level set at *P* < 0.001.

**Results:**

The results indicated a change in the molecular weight of different lens crystalline accompanied with changes in protein backbone structure. These changes increased for the groups exposed to IR for 10 minutes. Moreover, the activity of Na^+^-K^+^ ATPase significantly decreased for all groups.

**Conclusions:**

The protein of eye lens is very sensitive to IR radiation which is hazardous and may lead to cataract.

A variety of optical and electro-optical instruments are used for both diagnostic and therapeutic applications to the human eye. These generally expose ocular structures to optical radiations like infrared (IR) radiation.[1] The risk of developing cataract is more for workers who deal with hot materials such as molten glass or steel, due to exposure to IR radiation.[2] Ronald *et al*.[3] indicated that IR causes breakdown of the blood–aqueous barrier in rabbits. The relation between IR and cataract was studied by Okuno[4] who found that exposure to intense optical radiation led to the development of IR cataract in the work place. The epidemiological studies conducted by Sisto *et al*.[5] found a correlation between cataractogenesis and work with fused glass and metals. Zuclich *et al*.[6] summarized the results of a series of infrared laser-induced ocular damage that includes cornea, lens and retina. The authors concluded that the maximum permissible exposure limit should be considered. In another study by Vincelette *et al*.,[7] explaining laser–tissue effects from near-infrared radiation in the eye, there were evidences suggesting the presence of thermal lensing in ocular media.

According to the International Commission on Non-Ionizing Radiation Protection (ICNIRP)[8] statement, the IR energy from IR-A and IR-B poses a risk to the human eye. The penetration depth of these IR bands varies between 1.2 and 3 µm; therefore, the cornea, lens and retina can be damaged due to thermal effects associated with IR exposure. The aversion response normally limits the duration of exposure to less than 0.25–10 seconds. This protects the eye against thermal injury from sources such as the sun, incandescent lamps, and radiation emitted by hot objects. The IR radiation that is absorbed by the anterior segment (the cornea, aqueous, and lens) can produce clouding of the cornea and lens when the corresponding thresholds are exceeded. Exposure limits are set to protect both against acute as well as chronic exposure. Workers in hot environments, exposed to IR, developed lenticular opacities due to IR irradiance in the order of 80–400 mW/cm^2^ on a daily basis for 10–15 years.[9] Pitts and Cullen[10] showed that the threshold exposures for acute lenticular changes caused by IR-A were of the order of 5 kJ/cm^2^ for exposure durations of the order of an hour or longer and the threshold irradiances for damage were at least 4 W/cm^2^. The ICNIRP commission therefore recommended that to avoid the thermal injury of the cornea and the possible cataractogenesis, IR exposure (770 nm–3 µm) should be limited to 10 mW/cm^2^ for lengthy exposures (> 1000 seconds), and to 1.8 *t*^–3/4^ W/cm^2^ for shorter exposure durations. In cold environments, these limits may be increased to 40 mW/cm^2^ at 0°C and for special application, for reasons of comfort, this limit is approximately 30 mW/cm^2^ at 10°C.

In another study in which the heat transport within both the human and rabbit’s eyes was calculated, the calculated ocular temperature was rapidly increasing with the exposure time for the first 2 minutes, then gradually leveled off and reached the maximum within approximately 5 minutes. Furthermore, it takes several minutes for the eye to cool down after the exposure ceases.[11,12]

The present work was aimed to explore the molecular structure backbone of rabbit’s crystalline lens after exposure to IR irradiance of 0.2 W/cm^2^ for a period of 5 or 10 minutes, using infrared spectroscopy and electrophoresis. On the other hand, the activity of the Na^+^-K^+^ ATPase enzyme of lens membrane was monitored as a function of the IR irradiation time.

## Materials and Methods

Fifteen healthy New Zealand rabbits of either sex, weighing 2–2.5 kg, were used for this study. The animals were divided into three groups; one of them served as control. The other two groups were exposed to IR for 5 or 10 minutes. Animals from these two irradiated groups were subdivided into two subgroups; one of them was decapitated directly after IR exposure, while the other subgroup was decapitated 1 hour post exposure.

IR was delivered from a General Electric Lamp, model 250R 50/10, placed 20 cm from the rabbit and aimed at each eye. The lamp was calibrated at the Photometry Department, National Institute of standards, Giza, Egypt. The wavelengths emitted from the anterior surface of the IR lamp as provided by General Electric Lighting Division (Cleveland, OH, USA)[3] were 0.34–0.4 µm (UV light), 0.4–0.76 µm (visible light), 0.76–3.0 µm (IR-A, IR-B light 83%) and 3.0–7.0 µm (IR-C 10%). The total IR percentage emitted was 93%. The irradiance of the IR lamp detected at 20 cm was 0.2 W/cm^2^. The heat flux reaching the rabbit cornea after 5 minute IR exposure was 44 J/cm^2^; consequently, the heat flux reaching the cornea after 10 minutes was calculated and found to be 88 J/cm^2^.

The lenses were removed from the eye and their capsules were removed carefully. Each lens capsule was weighed in a separate container, and then homogenized in extraction medium [0.32 M sucrose, 1 mM ethylenediaminetetraacetic acid (EDTA) and 0.15% deoxycholic acid]. Na^+^-K^+^ ATPase measurement was carried out on the lens membrane by the method of Bowler and Tirri.[13]

The lenses without their capsules were weighed, homogenized separately in de-ionized water and centrifuged at 16,000 rpm to extract soluble lens proteins and then stored at –20°C for the following measurements.

Total proteins in the soluble part of crystalline lens were determined by the method of Lowry *et al*.[14] This method depends on preliminary treatment of proteins with an alkaline copper reagent followed by foline-phenol reagent. The developing color (measured spectrophotometrically at 750 nm) for different proteins depends on tyrosine, tryptophan content and the sequence of various amino acids with functional side groups, especially, histidine, arginine and glutamic acid.

Soluble lens proteins were separated according to their molecular weights by sodium dodecyl sulfate-polyacrylamide gel electrophoresis (SDS-PAGE) according to Laemmli,[15] using 5% stacking gel and 12% separating gel. The data were represented graphically with an automatic scanner (model R-112, manufactured by Beckman Coulter, CA, USA).

Fourier transformation infrared (FTIR) spectra of lyophilized soluble lens proteins were recorded on JASCO FTIR 430 spectrometer (JASCO Corporation, Tokyo, Japan) in the range 4000–400 cm^–1^. Measurements were made with an IR cell (KBr windows) according to Lamba *et al*.[16] The resolution was 2.0 cm^–1^ and all the spectra were recorded at physiological temperature. The instrument was operated under continuous N_2_ gas to reduce the effects of atmospheric CO_2_ and water vapor.

Data were presented as the mean ± SD. To determine the significance difference between the groups, analysis of variance (ANOVA) procedure was used followed by student’s *t*-test, where commercially available statistical software package, SPSS-11 for windows, was used. The significance level was set at *P* < 0.001.[17]

## Results

Table 1 gives the total soluble lens proteins of control rabbits and those exposed to IR radiation. The mean value of the control lens was 290.8 ± 3.8 mg/g wet wt. The exposed groups showed significant decrease, with an average value of 256.3 ± 15.7 mg/g wet wt. for both the groups exposed to IR radiation for 5 minutes. On the other hand, the average value of the groups exposed to IR for 10 minutes was 222.6 ± 18.5 mg/g wet wt. Na^+^-K^+^ ATPase activity [Table 1] shows a similar behavior as the total lens proteins but with different significant levels. For normal lens membrane, the enzyme activity was 48.2 ± 3.2 µMpi/hour/g wet wt. After exposure of rabbit eyes to IR radiation for 5 minutes, the enzyme activity was decreased for both the groups (*P* < 0.01), with an average value of 35.8 ± 1.7 µMpi/hour/g wet wt. After exposure to IR for 10 minutes also, the enzyme activity for both the groups decreased, with an average value of 25.9 ± 1.5 µMpi/hour/g wet wt.

**Table 1:** Total soluble protein content of rabbit lens and activity of Na^+^-K^+^ ATPase of rabbit lens membrane for the different studied groups

Groups	Total soluble protein (mg/g wet wt.) mean ± SD	Na^+^-K^+^ ATPase activity (µMpi/hour/g wet wt.) mean ± SD
Control	290.8 ± 3.8	48.2 ± 3.2
5 minutes-direct	267.4 ± 3.9†	37.0 ± 3.2†
5 minutes-1 hour	245.2 ± 4.2†	34.7 ± 2.2†
10 minutes-direct	235.6 ± 3.7†	26.9 ± 3.9†
10 minutes-1 hour	209.5 ± 4.1†	24.9 ± 2.8†

† Statistically significant

**Figure 1:**
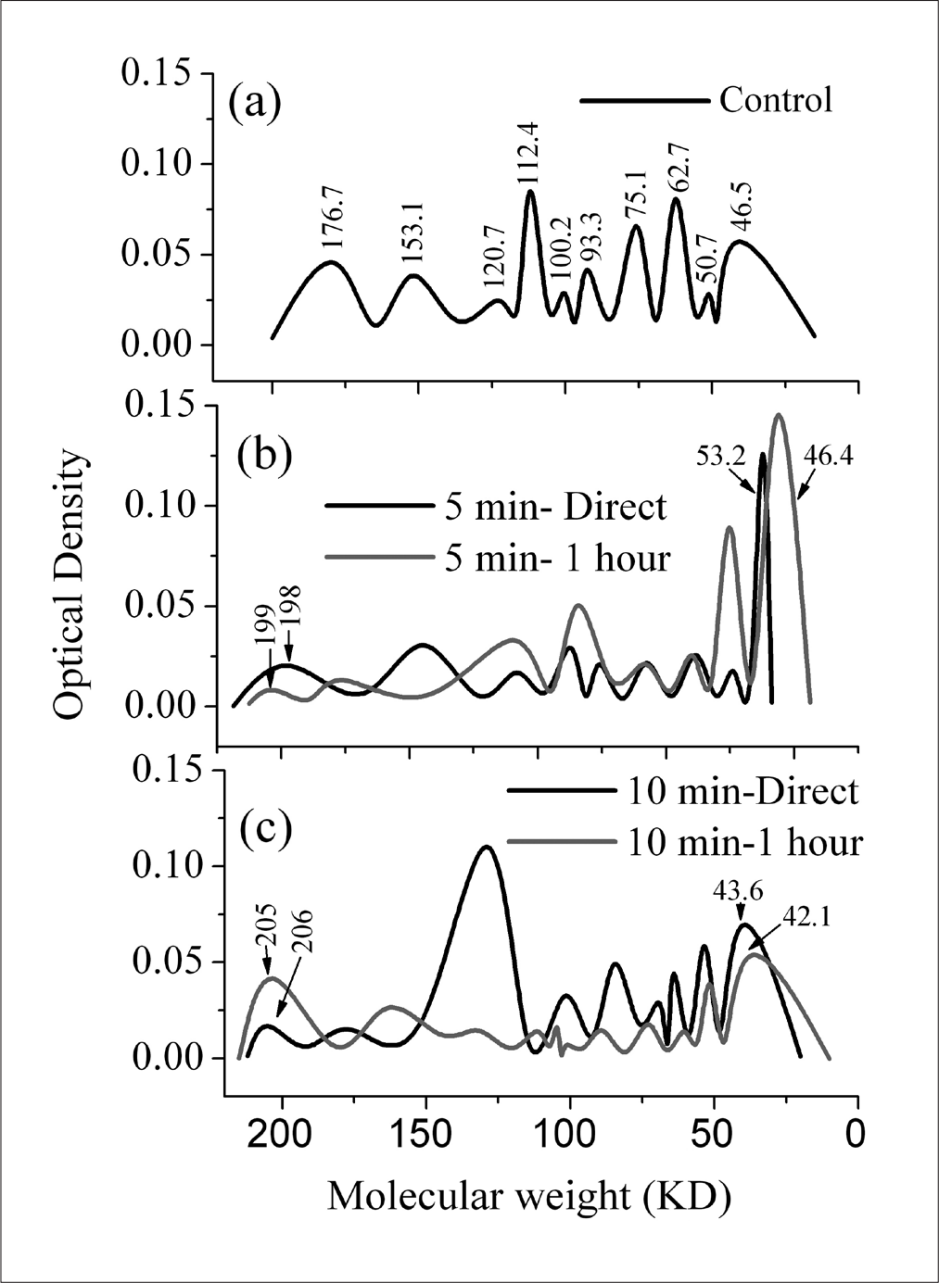
Electrophoretic profile of rabbit’s lens protein for (panel a) control group, (panel b) 5 minutes exposed groups to IR, directly decapitated and after 1 hour, and (panel c) 10 minutes exposed groups to IR, directly decapitated and after 1 hour

The electrophoretic patterns of the lens proteins for control [Fig. 1, panel a], 5 minutes exposure to IR direct and 1 hour post exposure [Fig. 1, panel b] and 10 minutes exposure to IR direct and 1 hour post exposure [Fig. 1, panel c] are shown.

In panel a of Fig. 1, the control was characterized by the presence of 10 peaks representing different soluble protein fractions with specific intensities and broadening, which cover the molecular weight range 177–47 kDa. In panel b of Fig. 1, the direct effect revealed the reduction of the soluble protein fractions to nine peaks which cover the molecular weight range 198–53 kDa. The maximum intensity was noticed for the last protein fraction (53 kDa). The reduction in the number of soluble protein fractions was pronounced after 1 hour of IR exposure (eight peaks). These peaks cover the molecular weight range 199–46 kDa. The maximum intensities were noticed for the last two fractions (60 and 46 kDa). Panel c of Fig. 1 shows the same result as that of panel b. The molecular weight range for 10 minute exposure-direct decapitation was 206–44 kDa, whereas after 1 hour of IR irradiation the molecular weight range was 205–42 kDa. The intensity of the peaks in the molecular weight range 126–44 kDa for the direct effect is higher relative to the corresponding peaks in 1 hour post exposure subgroup.

**Figure 2:**
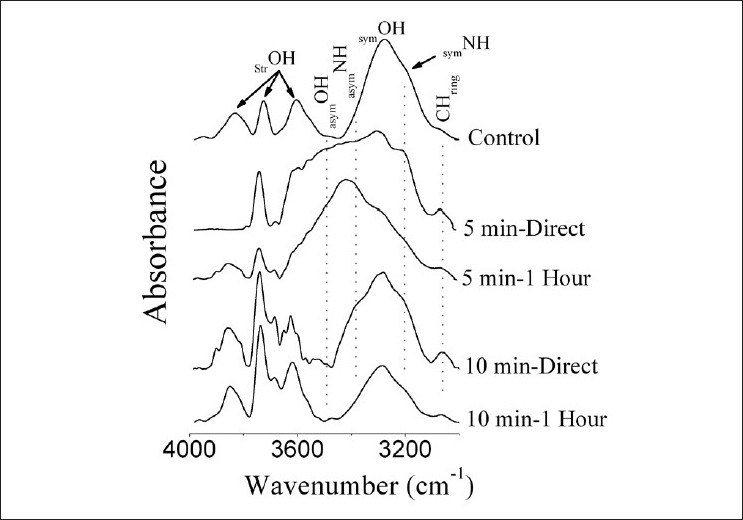
FTIR of soluble lens proteins for all studied groups in the NH-OH region (4000–3000 cm^–1^)

Fig. 2 shows the IR spectra of soluble lens proteins for control, 5 minute and 10 minute groups in the range 4000–3000 cm^–1^. The NH-OH region of the control pattern indicates the presence of six bands that centered nearly at 3850 cm^–1^ (_str_OH), 3739 cm^–1^ (_str_OH), 3616 cm^–1^ (_str_OH), 3288 cm^–1^ (_sym_OH), 3144 cm^–1^ (_sym_NH) and 3072 cm^–1^ (CH_ring_), respectively, as previously mentioned by Dovbeshko *et al*.[18] There were dramatic changes in the NH-OH pattern of all IR irradiated groups with different characteristics that differed according to the exposure period. For 5 minute irradiated groups, the changes can be noticed in the _str_OH region as reduced number of bands or as change in the contour; also, there was a distortion in the rest of bands where the contour was broad but not for the CH_ring_ where its intensities were increased. On the other hand, as the exposure time was increased to 10 minutes, the _str_OH was greatly affected where the number of bands was increased concomitant with the presence of _asym_OH vibration mode. Again, the intensity of CH_ring_ was increased. The _asym_NH mode can be noticed at 5 minutes-1 hour group as well as 10 minutes-direct one.

**Figure 3:**
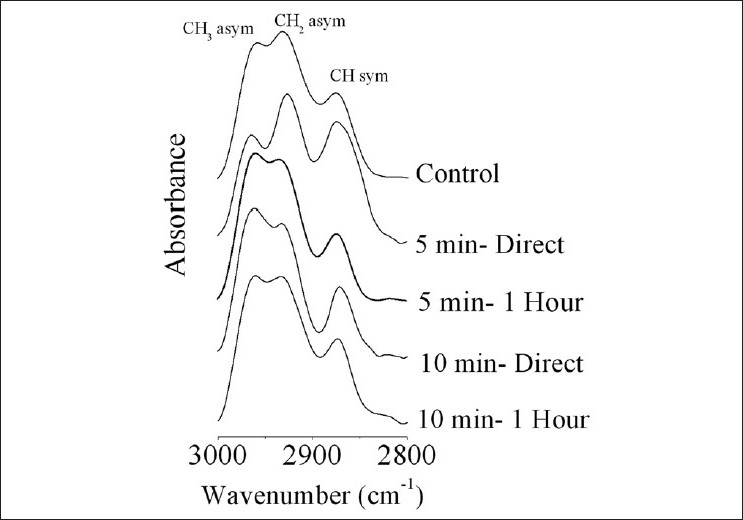
CH stretching region (3000–2800 cm^–1^) of soluble lens proteins studied by Fourier transform infrared for all the groups

Fig. 3 shows IR spectra of soluble lens proteins for control, 5 minute and 10 minute groups in the range 3000-2800 cm^–1^. This range characterizes the CH stretching region. The mean band position of control _asym_CH_3_ was 2967 ± 3 cm^–1^ and its bandwidth was 25 ± 3 cm^–1^, while for irradiated groups it was 2965 ± 3 cm^–1^ and 26 ± 2 cm^-1,^ respectively. The same phenomenon was noticed for the other two bands: _asym_CH_2_ (for control: 2930 ± 2 cm^–1^ and 43 ± 6 cm^–1^; for irradiated groups: 2929 ± 3 cm^–1^ and 40 ± 7 cm^–1^) and CH_sym_ (for control: 2973 ± 2 cm^–1^ and 32 ± 5 cm^–1^; for irradiated groups: 2971 ± 2 cm^–1^ and 31 ± 6 cm^–1^), i.e., no changes in either the band position or the bandwidth.

**Figure 4:**
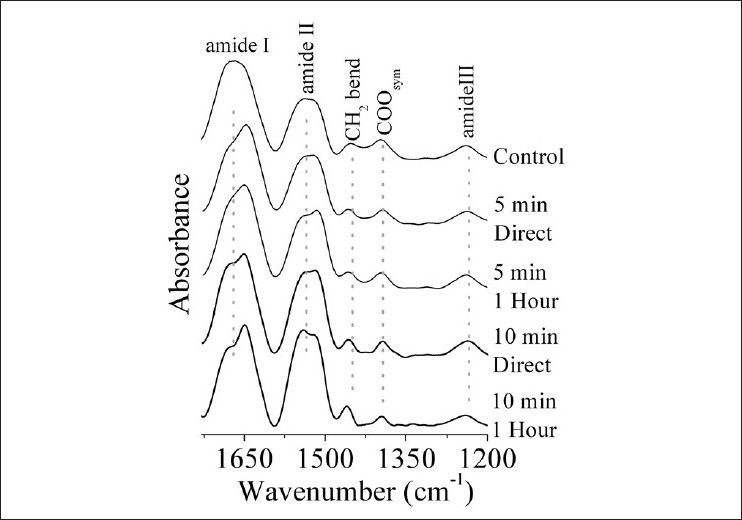
FTIR of soluble lens proteins for all the studied groups in the range 1700–1200 cm^–1^ (fingerprint region)

Fig. 4 shows the fingerprint region for all groups involved in this study. The interesting observation in this figure is the splitting of amide I band and amide II band into two components each. The mean vibrational frequency of control amide I was 1668 ± 1 cm^–1^. In the same context, the two components that were noticed in amide I region of irradiated groups had a mean frequency of 1680 ± 1 cm^–1^ and 1650 ± 2 cm^–1^, respectively. On the other hand, for amide II region, the mean vibrational frequency of the two bands in irradiated groups was 1543 ± 2 cm^–1^ and 1618 ± 2 cm^–1^, while in the control pattern this band was centered on 1533 ± 2 cm^–1^. The intensity of CH_2_ bending mode was increased for irradiated groups for 10 minute rather than 5 minute groups. COO_sym_ band was characterized by change in its contour at the 10 minutes-1 hour group. The frequency of amide III band was increased from 1237 ± 1 cm^–1^ in the control to 1241 ± 1 cm^–1^ in the IR irradiated groups. No change in the band intensity or band width was found for 5 minute irradiated groups. As the exposure time was increased to 10 minutes, the intensity of amide III band fluctuated, i.e., increased for the direct effect and reduced for the delayed 1 hour group. The bandwidth was reduced relative to the control in the direct studied group. The previous band assignment was based on that described by Toyran *et al*.[19]

## Discussion

Exposure to IR radiation may cause the corneal opacity, burns on the retina, miosis, breakdown of blood–aqueous barrier and delayed cataract.[20–23] The present study is an attempt to investigate the effect of IR radiation with different exposure times (5 and 10 minutes) on the molecular structure of the soluble lens proteins.

The lens proteins are markedly decreased after exposure to IR radiation for all the studied groups. This decrease is directly proportional to the time of exposure. Also, the decrease of total lens proteins is more pronounced in the group consisting of animals which were decapitated after 1 hour of exposure. This decrease may be due to cataract formation. When IR radiation is incident on the eye, it is absorbed by the cornea and converted into heat which is then conducted to the lens and induces cataract.[4] The changes in the lens crystalline, evidenced by SDS-PAGE, are given in Fig. 1. It is clear that there were changes in the molecular weight, electrophoretic mobility and intensity of different peaks representing different crystalline fractions. The obtained changes in the molecular weight of lens proteins are responsible for the decrease in the concentration of soluble lens proteins of all the studied groups and this may be a characteristic of cataract. Michael *et al*.[24] concluded that during cataract formation, the decrease of biosynthesis of lens crystalline was followed by their aggregations which then led to opacity of the lens.

Na^+^-K^+^ ATPase is an enzyme that has been found to play a major role in ionic transport through cell membrane. Decreased levels of Na^+^-K^+^ ATPase activity have been detected after exposure to IR radiation. This decrease may be due to the disturbance of lens cell membrane function, which leads to a decrease in active transport of nutrients and electrolytes from the aqueous into the lens. Kantorow *et al*.[25] and Delamere *et al*.[26] found that Na^+^-K^+^ ATPase has a lower activity in certain types of human cataract and also in a number of experimental cataracts.

The decrease in the total soluble proteins and the change in the molecular weight of lens proteins result from the conformational changes in protein secondary structure, which were noticed in the FTIR data. Chen *et al*.[27] recorded similar findings as ours, where compositional and conformational changes of lens proteins were associated with cataract formation. In the present study, the dramatic changes noticed in the NH-OH region [Fig. 2] indicate that the soluble lens protein was greatly influenced by IR radiation and that the magnitude of response was proportional to the exposure time. These changes indicate the presence of different interaction mechanisms between the soluble lens crystallines that function in the exposure time, i.e., there are two different environments. The vibrational motion of hydrocarbon chains of proteins is unaffected by these IR exposure periods [Fig. 3]. The soluble protein part of the rabbit’s lens was greatly affected by the IR irradiation periods of 5–10 minutes [Fig. 4]. Amide I band indicates that the soluble protein secondary structure was modified with the domination of β-turns structure (band at 1680 cm^–1^) and the α-helix one (band at 1652 cm^–1^). The presence of β-turns structure implies that protein becomes more folded and may tend to be aggregated. These findings were clearly confirmed by the observation of splitting of amide II band.

In conclusion, our FTIR findings provide information about the mechanism of protein structural changes induced by IR exposure periods of 5 or 10 minutes. IR exposure may participate in cataract formation. Furthermore, the alteration of the soluble lens protein secondary structure resulted in a reduction of the Na^+^-K^+^ ATPase activity.
